# The Social Horizon

**Published:** 1892-12-31

**Authors:** 


### THE PEOPLE'S NEEDS.

### THE SOCIAL HORIZON.

Under this title* a gentleman whose pity has been moved by the miseries of the London poor, sketches the course towards which he thinks municipal and national policy must and should move. As he tells us repeatedly that he is a journalist, and that it was in the course of his professional investigations he saw the sights which have moved him to pen and publish this volume, we are guilty of no discourtesy in defining this as a journalist's book. That is, it contains keen observation, vivid description, ready and contagious sympathy, the power of impressing the reader—a journalist's merits; combined with a deficiency in historical knowledge and a weakness in philosophic inference from facts, which are the journalist's commonest faults. Things are wrong in this world of ours, but our author has "a pattern on his thumb" by which all may be put right. Economic philosophers do not admire it; that is, we are told, because they are the selfish sycophants of a yet more selfish dominant class who love to sweat and grind the poor if thereby they may themselves be enriched. Clergymen, even those who dwell among the poor, do not invariably clasp the grimy hands of the street-corner orator who draws this pattern for the benefit of the mob, but our journalist does not give them credit for knowing something of the people around them, and because of this knowledge doubting if the orator's roseate visions will ever be fulfilled; no, they hold back because they are not true followers of Him in whom we are to see nothing but the Carpenter of Nazareth. They are condemned for preaching that moral regeneration must precede physical well-being, though that same Carpenter said, "Seek ye first the kingdom of God and His righteousness," and philosophers who would admit no inspiration in the saying, but have studied carefully the phenomena of human life, agree that, to be permanent, material well-being must have a moral basis.

This book is so obviously well-intentioned that it is unpleasant to be forced to criticise it harshly; and, in so far as its leading suggestion is concerned—which we take to be the municipalisation or nationalisation of the various modes of locomotion—we have no harsh criticism to offer. The thing is, to say the least of it, worth trying. But, if we do not see how it can be worked so as to get rid of poverty and overwork, if we do not share our author's views on the elimination of capitalists, if we suggest that there is a bottom to the national purse, we ask him not to describe us so promptly and recklessly as he seems inclined to do as a compound of

* "The Social Horizon." By the author of "Life in Our Villages." (London: Swan Sonnenschein, and Co.)

knave and fool. Even the old orthodox political economists whom he hates so much had, like himself, honest intention in their theories and deductions.

The author of "The Social Horizon" condemns them for having, in reckoning up the wealth of a community, judged it by the total amount, irrespective of equality of distribution. The individualistic, the *laissez-faire* system, is, he says, adapted to make the rich ever richer, the total wealth of any community increasingly greater, while the poor are ground in the dust under the juggernaut car of capital. Now let us look at the facts. We learn from Mr. John Rae's book on "Contemporary Socialism," which is a full, thoughtful, and complete text-book of the subject, that according to the statistics compiled by Gregory King in 1688 the average income of a working-class family at that time was £12 12s. per annum; the average income of such a family now is £81. "In 1688 74 per cent. of the whole population belonged to the working class, and they earned collectively 26 per cent. of the entire income of the country; in 1867 80 per cent. of the whole population belonged to the working class, and they earned collectively 40 per cent. of the entire income of the country. Their share of the population has increased 6 per cent.; their share of the income 14 per cent." Hitherto, then, it does not seem that the individualist system has been to the disadvantage of the working man; his income has not merely increased absolutely, but relatively to the incomes of other classes it has increased more rapidly.

How has this come about under the condemned individualist régime? We do not mean to say that the capitalist is of his own free will a generous being who gives an increasing share of his wealth to the working man. No; he is not inevitably a philanthropist, and trades unions have forced him to give more and more to his workers till the limit of wages is no longer fixed at the least the workman can afford to take, but at the utmost an employer can afford to give. With increasing wages the working classes have adopted a higher scale of living, which in return has helped, directly and indirectly, to keep wages up. Directly, by making any attempt to lower them arouse discontent and rebellion, culminating in strikes; indirectly, by making the prudent refrain from marriage until they had fair assurance of an income which would permit them to obtain the comforts, now become necessaries, usual to their class. But, besides this which he has given grudgingly and unwillingly—we have no wish to idealise him—the capitalist has of his own free will put money into the pockets of the working class. How? Thus—his main object is to obtain, as he will tell you, a profitable investment for his money. To this end he develops new industries, seeks out new markets, explores and exploits the East and the West; and in all his doings gives work and wages. It is all for his own benefit, perhaps, but none the less it benefits others as well. The capitalist may not be a beautiful factor in our industrial development, but he has undeniably been a useful one.

If he is destroyed he may, indeed, be replaced by co-operation, by municipal and national Socialism. But a mass does not move so quickly as an individual. Co-operative bodies may go on steadily on ascertained lines; but they seldom have the energy, the enterprise, the resourcefulness of the individual who feels that the making or marring of his fortune lies with himself. Had the State been the sole employer of labour throughout this century, one may doubt if any, short of the pauper class, would have been so well off as they are to-day. The "orthodox political economists," in speaking of national wealth, are not speaking so exclusively of capitalists and millionaires as our author thinks. It is not only those who ride in carriages and fare sumptuously every day that profit by the labours of others. "The man who works with pick and shovel makes, according to Mr. Mulhall's estimate" (again we quote from Mr. Rae's book), "£30 a year now, while he only made £12 a year in 1800, when bread was about twice as dear. . . . How did he come to get these better wages? It was not on account of an increase in his own production; it was on account of the general increase in the productivity of all labour around about him. The great improvement in industrial processes has brought in more plentiful times, and he shares in the general plenty, though he may not have directly contributed to its production." It is well to consider this point in contemplating a change which is to kill the capitalist and all his works.

It is generally admitted, indeed, that railways, tramways, omnibuses, and the like may conveniently be worked by the State, but when, through this, our author sees on the social horizon a great diminution of the unemployed, we fear he walks by faith and not by sight. The omnibus drivers and conductors of London are overworked and underpaid, he says: why not add to their number 10,000 men taken from the unemployed, give all a better wage, and clothe all in a comfortable uniform (the making of which would employ a large number of East-end seamstresses). If this were done profits might disappear, and capital would refuse to put money into omnibus companies. Then, says our author, let us municipalise them, and no profits will be needed. Agreed, though capital in some form, whether drawn from the rates or not, would be required to purchase carriages, horses, &c. But if with the increased expenditure suggested the omnibuses can only be run at a loss, what then? Municipalities can no more afford to lose than private individuals, and must make up the deficit either by an increased rate, against which those ratepayers who do not ride in omnibuses would protest, or, more probably, by an increased fare. But there are many—we speak feelingly, having been numbered among them—to whom the difference of a penny in a omnibus fare means just the difference between taking it or not. The increased price lessens the demand, and no more money is taken than before. We have taken the example our author gives, but the principle holds good of many branches of trade. There are many commodities for which a demand exists only because they are cheap, and which even a slight increase of cost in production would annihilate altogether. And collectivism would not inevitably change this; money would not be forthcoming to run any industry at a loss, or if it was, the taxpayers would, like the Cornishmen, "know the reason why." And, as has been said, enterprise would tend to fade and fail. Our author, indeed, declares with a sort of triumph that the heads of Government departments are often willing to make experiments, if only the Treasury would let them! But the Treasury refuses the necessary funds; and if it does so now in small issues would it be more reckless if it controlled all the industries of the nation? As custodian of the national purse, trustee of money that represents the labours of every citizen, it dares not, then or now, unless it is prepared to face the problem of national bankruptcy.

We have dealt at some length with this book because we regard it as a popular utterance of a popular feeling. If we have condemned its schemes as Utopian and its accusations as unjust, we desire nevertheless to express our sympathy with the feeling which inspired it, and the tenderness for the suffering poor which leavens every page.

